# Synthesis of szentiamide, a depsipeptide from entomopathogenic *Xenorhabdus szentirmaii* with activity against *Plasmodium falciparum*

**DOI:** 10.3762/bjoc.8.60

**Published:** 2012-04-11

**Authors:** Friederike I Nollmann, Andrea Dowling, Marcel Kaiser, Klaus Deckmann, Sabine Grösch, Richard ffrench-Constant, Helge B Bode

**Affiliations:** 1 Stiftungsprofessur für Molekulare Biotechnologie, Institut für Molekulare Biowissenschaften, Goethe Universität Frankfurt, Max-von-Laue-Straße 9, D-60438 Frankfurt a. M., Germany; 2 Biosciences, University of Exeter in Cornwall, Tremough Campus, Penryn, Cornwall TR10 9EZ, United Kingdom; 3 Swiss Tropical and Public Health Institute, Parasite Chemotherapy, Socinstr. 57, P.O. Box, CH-4002 Basel, Switzerland; 4 Institut für klinische Pharmakologie, Uniklinik Frankfurt, Theodor-Stern-Kai 7, D-60590 Frankfurt a. M., Germany

**Keywords:** cyclic depsipeptide, esterification, natural product, szentiamide, *Xenorhabdus*

## Abstract

The synthesis of the recently characterized depsipeptide szentiamide (**1**), which is produced by the entomopathogenic bacterium *Xenorhabdus szentirmaii*, is described. Whereas no biological activity was previously identified for **1**, the material derived from the efficient synthesis enabled additional bioactivity tests leading to the identification of a notable activity against insect cells and *Plasmodium falciparum*, the causative agent of malaria.

## Introduction

**Figure 1:**
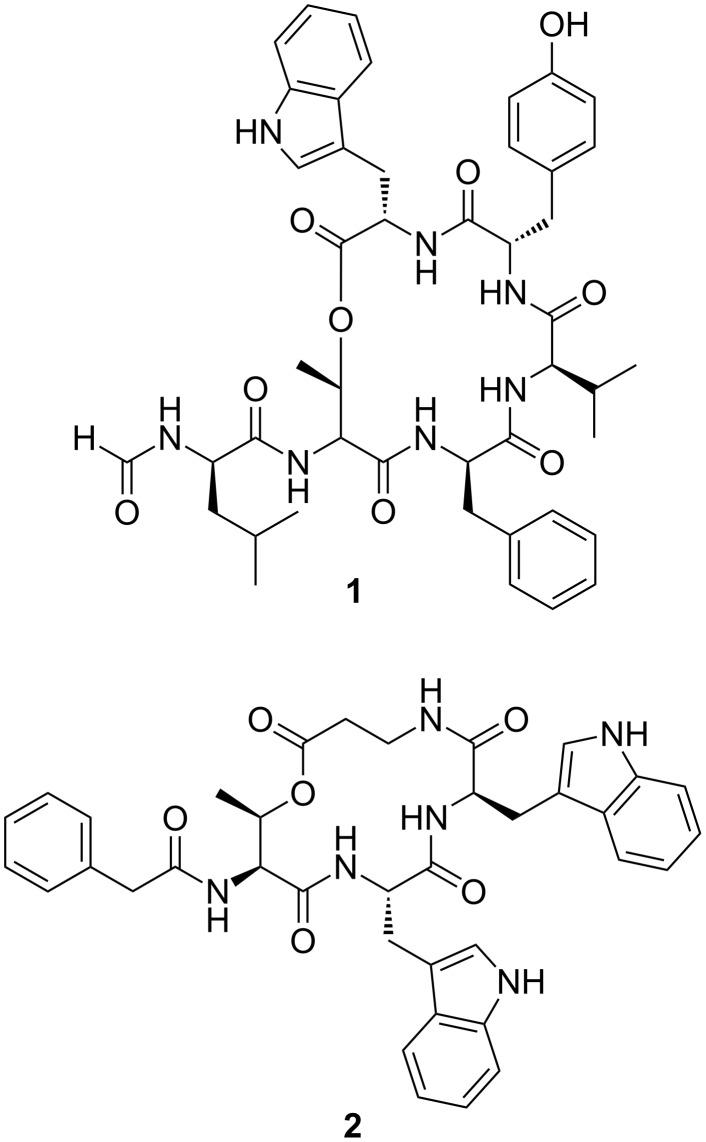
Structure of depsipeptides szentiamide (**1**) [12] and xenematide (**2**) [8] identified in *Xenorhabdus* strains.

Bacteria of the genus *Xenorhabdus* live in symbiosis with nematodes of the genus *Steinernema* and together they form an entomopathogenic complex that can infect and kill several insect larvae. During this complex life cycle the bacteria produce secondary metabolites, which may be involved in and/or may be required for different stages of this life cycle, including the symbiotic stage (towards the nematode) or pathogenic stage (towards the insect prey) [1–3]. Until three years ago, the natural products extracted from *Xenorhabdus* and its close neighbour *Photorhabdus* were only low-molecular-weight compounds with UV chromophores (e.g., isopropylstilbenes [4], anthraquinones [4], or xenorhabdines [5]. However, bioactivity-based or MS-based screening of crude extracts and culture supernatants led to the identification of larger compounds, such as the PAX peptides [6], the xenortides [7], xenematide [8] and the GameXPeptides [9]. Analysis of the genome sequences of the fully sequenced members of *Xenorhabdus* and *Photorhabdus* [10–11] has revealed that several additional compounds and especially even much larger compounds await isolation and structure elucidation. Recently, szentiamide (**1**) has been isolated, representing only the second depsipeptide (Figure 1) from these bacteria [12]. It is composed of six amino acids having a formylated *N*-terminus and raised our interest as it is produced by *X. szentirmaii*, whose crude extract shows a very high biological activity in several different bioassays (unpublished data). Nevertheless, no bioactivity has been described for **1** so far. Since we believe that **1** must have a biological function that is simply awaiting its identification, and since the peptide can only be found in small amounts when *X. szentirmaii* is grown in Luria–Bertani media, we wanted to synthesize it and make it accessible for additional bioactivity tests.

## Results and Discussion

### Synthesis

**Scheme 1:**
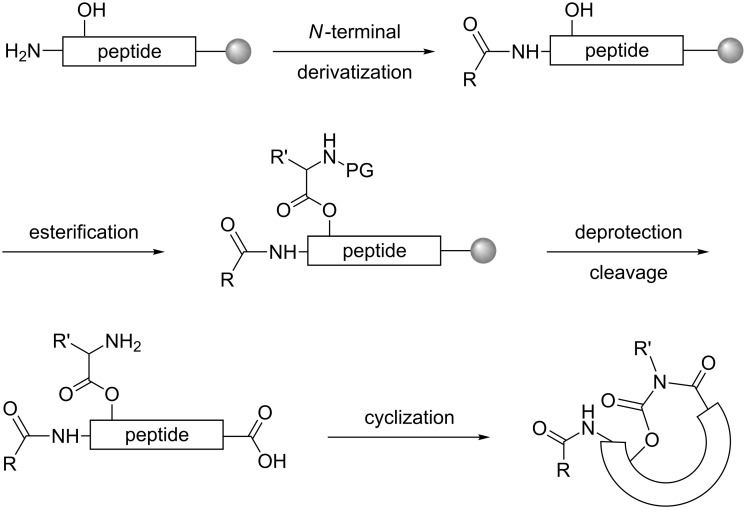
Overview of the synthetic strategy.

As previous syntheses of depsipeptides showed that lactamization was preferred over lactonization [13–14], the synthesis of **1** was performed as follows: briefly, the linear peptide was synthesized using solid-phase peptide synthesis, followed by esterification and subsequent cleavage from the resin, deprotection and cyclization to yield **1**, assisted by microwave irradiation at every stage with the exception of the esterification (Scheme 1).

**Scheme 2:**
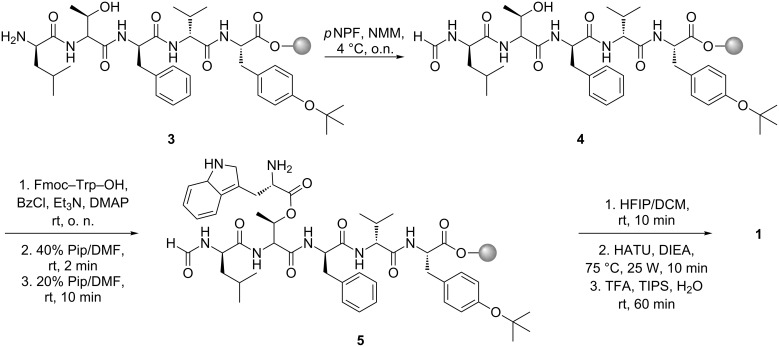
Synthesis of compound **1**.

In detail, a preloaded 2-chlorotrityl chloride resin was used in order to avoid nonspecific interaction in the cyclization process. Prior to the synthesis, the resin was reactivated [15] and then loaded with Fmoc-L-Tyr(*t*-Bu)-OH [16], followed by the build-up of the linear sequence **3** (Scheme 2) with *O*-benzotriazole-*N,N,N’,N’*-tetramethyluronium hexafluorophosphate (HBTU) in dimethylformamide (DMF) and *N*,*N*-diisopropylethylamine (DIEA) in *N*-methylpyrrolidone (NMP), assisted by microwave irradiation. After the final Fmoc-deprotection with 20% piperidine in DMF, the *N*-terminus was formylated with *para*-nitrophenyl formate (*p*NPF) in the presence of *N*-methylmorpholine (NMM) at 4 °C, affording the synthetic intermediate **4**.

**Figure 2:**
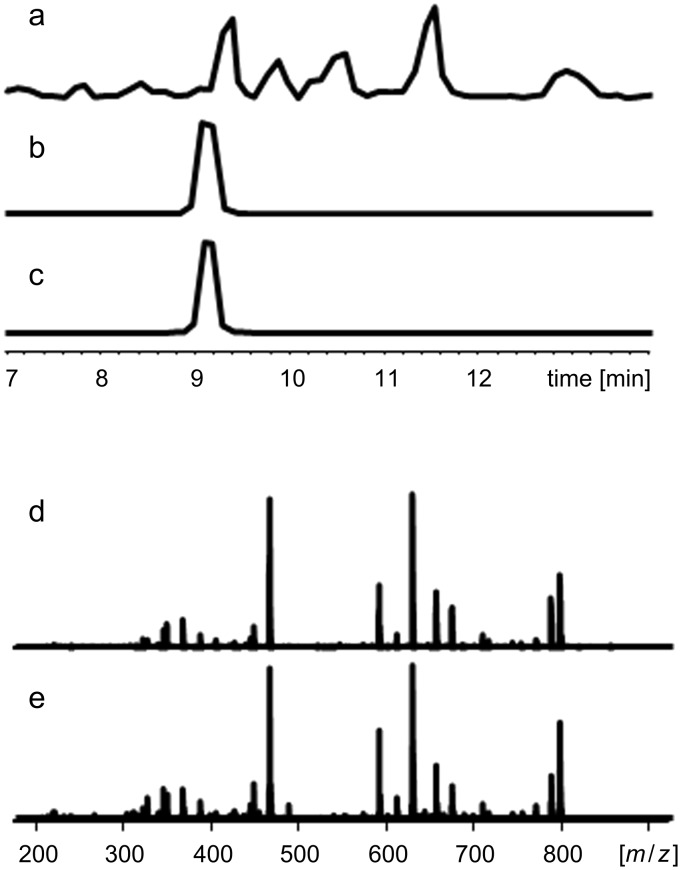
HPLC–MS data of an XAD-extract of *X. szentirmaii* (a; base-peak chromatogram), the natural **1** (b; extracted-ion chromatogram) and synthetic **1** (c; extracted-ion chromatogram) with their MS^2^-spectra (d, natural **1**, and e, synthetic **1**).

The attempts to form the ester bond by using catalytic amounts of 4-dimethylaminopyridine (DMAP) with 1-ethyl-3-(3-dimethylaminopropyl)carbodiimide (EDC), DMAP together with *N,N′*-diisopropylcarbodiimide (DIC), or a mixture of the DMAP hydrochloride and DMAP together with DIC all turned out to be unsuccessful. However, we were then able to establish the ester bond in **5** using modified Yamaguchi conditions [17]. Subsequently, the Fmoc-protecting group was removed at room temperature, and the peptide was cleaved from the resin with 3% hexafluoroisopropanol (HFIP) in dichloromethane (DCM) in order to preserve the side-chain protecting group. Following this, the peptide was cyclized in solution by using *O*-(7-azabenzotriazol-1-yl)-*N,N,N′,N′-*tetramethyluronium hexafluorophosphate (HATU) and DIEA in DMF, assisted by microwave irradiation. Afterwards, the remaining side-chain protecting group was removed by incubation with a common cleavage cocktail for 60 min at room temperature to give the crude product **1**, which was purified by preparative reversed-phase HPLC (yield: 14% from the resin (B = 0.84 mmol/g)). In order to compare the synthetic to the natural product we isolated **1** from *Xenorhabdus szentirmaii* DSM 16338 as described previously [12]. Briefly, the strain was cultivated in a shake flask containing Luria–Bertani media and 2% Amberlite XAD-16 adsorber resin. After cultivation for three days at 30 °C, the resin was collected and the bound substances were eluted with methanol (MeOH) repeatedly. The resulting brown, oily crude extract was fractionated by normal-phase flash chromatography, followed by the isolation of compound **1** by preparative reversed-phase HPLC. In contrast to already published data [12] we were able to isolate 26.8 mg from a 2 L culture, which corresponds to a yield of 0.015 % (m/v). Thus, the addition of Amberlite XAD-16 adsorber resin led to a 150-fold increase of the production of **1** in comparison to cultures cultivated without XAD-16 [12]. In fact the productivity was even higher, since it was obtained from a three-day instead of the described eight-day cultivation of *X. szentirmaii*. Comparison of the LC–MS (Figure 2b and c) and NMR data (Figure S1, S2 and Table S1 in Supporting Information File 1) proved the synthetic **1** to be identical to the natural product.

### Biological testing

The cyclic depsipeptide **1** was tested against different Gram-positive (*Micrococcus luteus, Bacillus subtilis, Staphylococcus aureus*) and Gram-negative (*Escherichia coli, Pseudomonas aeroginosa*) bacteria, as well as yeast (*Candida albicans, Saccharomyces cerivisiae*). However, consistent with the published data [12], no antibacterial or antifungal activity was detected. Additionally, the peptide **1** was tested against several parasites (*Trypanosoma brucei rhodesiense, Trypanosoma cruzi, Leishmania donovani, Plasmodium falciparum*) being the causative agents of the neglected tropical diseases [18] sleeping sickness, leishmaniasis and malaria. Interestingly, a good activity against the malaria-causing parasite *P. falciparum* (IC_50_ = 0.995 µg/mL) was observed, but only a 50- to 80-fold weaker cytotoxicity (L6 cells, IC_50_ = 57.4 µg/mL and HeLa cells, IC_50_ > 80 µg/mL). Only a weak activity was observed against *T. b. rhodesiense* and *L. donovani* with IC_50_ = 10.0 µg/mL and IC_50_ = 11.0 µg/mL, respectively. Additionally, we also tested **1** against hemocytes of *Galleria mellonella* and could detect a LD_50_ value of 59.7 µg/mL.

## Conclusion

The establishment of an efficient synthesis route for the depsipeptide szentiamide (**1**) from *X. szentirmaii* revealed its biological activity against insect cells and protists such as *P. falciparum*. The rationale behind this bioactivity may be that **1** adds to the overall insecticidal activity of *Xenorhabdus* bacteria. Protists such as amoeba are common soil inhabitants, which may feed on the dead insect cadaver. Thus, compounds such as **1** may protect the insect cadaver against these food competitors and “accidentally” may also target pathogenic protists such as *Plasmodium*, which is a global human threat. Therefore, the bioactivity of **1** revealed in this study highlights the potential of *Xenorhabdus* bacteria as producers of bioactive natural products and the importance of a broad bioactivity testing of isolated compounds in order to find a biological activity and thus a biological function of such natural products. Work in the Bode lab currently concentrates on the identification of the mode of action of **1** in insects and protists in order to also understand its molecular function.

## Experimental

### Synthesis

Unless otherwise stated, we used the chemicals in their highest available purity. The progress of the synthesis was monitored with MALDI–MS as well as RP-UPLC coupled with ESI–MS.

**Solid-phase peptide synthesis.** The linear sequence was synthesized on a preloaded 2-chlorotrityl chloride resin (Carbolution Chemicals, Germany) on a 50 μmol scale with the Discover CEM System by using standard 9-fluorenylmethoxycarbonyl/*tert*-butyl (Fmoc/*t-*Bu) chemistry. An amount of 6 equiv of amino-acid derivatives (>98%; Iris Biotech, Germany/Carbolution Chemicals; *c* = 0.2 mol/L) was activated in situ with 5 equiv *O*-benzotriazole-*N,N,N′,N′*-tetramethyluronium hexafluorophosphate (HBTU; Iris Biotech) in dimethylformamide (DMF; Acros Organics, Belgium; *c* = 0.5 mol/L) in the presence of 10 equiv *N*,*N*-diisopropylethylamine (DIEA; Novabiochem, Darmstadt, Germany) in *N*-methylpyrrolidone (NMP; VWR, Germany; *c* = 2 mol/L). Fmoc protecting groups were cleaved with 20% piperidine in DMF by using microwave irradiation as well.

**Formylation.** The free *N*-terminus was formylated with 5 equiv *para*-nitrophenyl formate (*p*NPF; Sigma Aldrich, Germany) and 3 equiv *N*-methylmorpholine (NMM; Sigma Aldrich, Germany) in DMF (*c* = 12.5 mmol/L) at 4 °C over night.

**Ester bond formation.** The depsipeptide bond was formed by using 20 equiv Fmoc-protected amino acid, 20 equiv benzoyl chloride (BzCl, Sigma Aldrich, Germany) and 40 equiv (Et_3_N, Sigma Aldrich, Germany) in DCM (*c* = 62.5 mmol/L) first at 0 °C then with warming to room temperature overnight. After the quantitative reaction, the Fmoc-protecting group was cleaved by using 40% piperidine in DMF for 2 min and then 20% piperidine in DMF for 10 min at room temperature.

**Cleavage and cyclization.** The protected branched peptide was cleaved with 3% hexafluoroisopropanol (HFIP; Carbolution Chemicals, Germany) in dichloromethane (DCM; VWR, Germany) and cyclized in solution (20 min, 25 W; 75 °C) by using *O*-(7-azabenzotriazol-1-yl)-*N,N,N′,N′-*tetramethyluronium hexafluorophosphate (HATU; Carbolution Chemicals, Germany) and DIEA in DMF (*c* = 4 mmol/L). The cyclized product was fully deprotected by incubation with 95% trifluoroacetic acid (TFA; Iris Biotech, Germany) and 2.5% triisopropylsilane (TIPS, Alfa Aesar, Germany) in deionized water at room temperature for at least 60 min. Then the cleavage cocktail was evaporated and the peptide dissolved in MeOH in order to purify it by HPLC–MS (Waters^®^ Purification^TM^ System, Waters Corporation, USA; Jupiter Proteo, Phenomenex, Germany). The purity was determined by RP-UPLC coupled with ESI–MS.

**Fermentation. ***Xenorhabdus szentirmaii* was cultivated at 30 °C and 280 rpm on a rotary shaker in two 5 L Erlenmeyer flasks each containing 1 L of Luria–Bertani (LB) broth (pH 7.0) and 2% (v/v) of XAD-16 (Sigma-Aldrich, Germany). These cultures were inoculated with 1% (v/v) of an 18 h preculture in the same medium without XAD-16. Cultures were harvested after three days, and XAD beads were separated from the supernatant by sieving.

**Isolation.** After washing with H_2_O the XAD beads where extracted with MeOH (2 × 50 mL), followed by concentration to dryness under reduced pressure, yielding a brown oily residue and amorphous precipitate. This was dissolved in 4 mL MeOH, centrifuged for 10 min and 13000 rpm at room temperature and the pure compound isolated from the supernatant by HPLC-MS.

### Biological testing

**Disk diffusion Test.** The nonpathogenic strains *E. coli* BL21, *M. luteus, B. subtilis* and *S. cerevisae* PK113 were cultured overnight at 30 °C in LB and YPD media. Agar plates were overlaid with an inoculum (turbidity equivalent to the optical density of 0.5 measured at 600 nm) of the different strains. Cellulose disks (100% cotton linter; Carl Roth, Karlsruhe, Germany) were loaded with 100 µg of the peptide. The dried disks were applied to the prepared agar plates and incubated for 24 h. Then the inhibition zones were measured following NCCLS criteria [19].

**Hemocyte cytotoxicity analysis** [20]. Last instar *Galleria mellonella* (Greater wax moth) larvae (Livefoods, UK) were anaesthetized by chilling on ice for 30 min. The larvae were surface sterilized with 70% ethanol before one of the first prolegs was excised with micro-scissors. Approximately 1.5 mL of out-flowing hemolymph was collected from the larvae directly into 10 mL chilled supplemented Graces Insect Medium (GIM) (Gibco, Invitrogen) and mixed rapidly by inversion. The hemocyte suspension was then centrifuged at 200 *g* for 5 min, the supernatant aspirated and the hemocyte pellet gently resuspended in 1 mL GIM before being made up to a final 10 mL dilution. The hemocyte suspension was arrayed into black microplates (Greiner microclear) and then incubated at 28 °C undisturbed for 60 min to allow the cells to settle and adhere. The monolayers were washed with GIM before the addition of GIM containing 100, 10 or 1 µg/mL of each of the compounds, which were co-incubated with the hemocytes for 4 h. Following incubation, the GIM and compounds mixture was aspirated and replaced with GIM containing 500 nM Mitotracker CMH_2_XRos for 45 min at 28 °C (Molecular Probes, Invitrogen). Hemocyte monolayers were washed with 1× PBS and fixed with 4% paraformaldehyde for 15 min before permeabilizing with 0.2% Triton X-100 in PBS for 10 min. Cells were stained with FITC-conjugated phalloidin and Hoechst 33258 and finally washed with PBS. The plate was imaged by using the IN Cell Analyzer 2000 (GE Healthcare) and analyzed with the IN Cell Analyzer 1000 Workstation software. Estimates of LD_50_ values were calculated by using the R statistical package [21].

**Cell viability assay. HeLa cells:** The water-soluble tetrazolium-1 salt (WST-1; Roche Diagnostics, Germany) was used to determine the cell viability after treatment of cells with the compounds. HeLa cells were seeded at a density of 3 × 10^3^ cells in 100 µL culture medium containing 10% FCS into 96-well microplates and incubated for 24 h at 37 °C. The medium was removed and HeLa cells were treated with increasing concentrations of the compound (10, 50 and 100 µM) or dimethyl sulfoxide. After 24 h, 10 µL of WST-1 reagent was added to each well and the cells were incubated for a further 90–150 min. The formation of the formazan was measured at 450 nm against a reference wavelength of 620 nm by using a 96-well spectrophotometric plate reader (SpectraFluor Plus, Tecan, Crailsheim, Germany). **L6-cells:** Assays were performed in 96-well microtiter plates, each well containing 100 μL of RPMI 1640 medium supplemented with 1% L-glutamine (200 mM) and 10% fetal bovine serum, and 4 × 10^4^ L6 cells (a primary cell line derived from rat skeletal myoblasts). Serial drug dilutions of seven three-fold dilution steps, covering a range from 90 to 0.123 μg/mL, were prepared. After 72 h of incubation, the plates were inspected under an inverted microscope to assure growth of the controls and sterile conditions, 10 μL of Alamar Blue solution was then added to each well and the plates were incubated for another 2 h. Then the plates were read with a Spectramax Gemini XS microplate fluorometer with an excitation wavelength of 536 nm and an emission wavelength of 588 nm. Data were analysed by using the microplate-reader software Softmax Pro.

**Activity testing against parasitic protozoa**. Bioactivity against the four protozoan parasites *P. falciparum* (NF54), *T. cruzi* (Tulahuen C4), *T. b. rhodesiense* (STIB900), and *L. donovani* (MHOM-ET-67/L82) was determined as previously described [22].

## Supporting Information

File 1 NMR-data of szentiamide (**1**).
