# Supplementary material for: Synthesis of szentiamide, a depsipeptide from entomopathogenic Xenorhabdus szentirmaii with activity against Plasmodium falciparum
Source: Beilstein J Org Chem. 2012 Apr 11;8:528–33. doi: 10.3762/bjoc.8.60 (PMC3343279; doi:10.3762/bjoc.8.60)
Supplement: File 1 — NMR-data of szentiamide (1). [file Beilstein_J_Org_Chem-08-528-s001.pdf]

Supporting Information

for

Synthesis of szentiamide, a depsipeptide from entomopathogenic *Xenorhabdus szentirmaii* with activity against *Plasmodium falciparum*

Friederike I. Nollmann[1], Andrea Dowling[2,§], Marcel Kaiser[3,§], Klaus Deckmann[4,§], Sabine Grösch[4,§], Richard ffrench-Constant[2,§], Helge B. Bode*[1]

Address: [1]Stiftungsprofessur für Molekulare Biotechnologie, Institut für Molekulare Biowissenschaften, Goethe Universität Frankfurt, Max-von-Laue-Straße 9, D-60438 Frankfurt a. M., Germany; [2]Biosciences, University of Exeter in Cornwall, Tremough Campus, Penryn, Cornwall TR10 9EZ, United Kingdom; [3]Swiss Tropical and Public Health Institute, Parasite Chemotherapy, Socinstr. 57, P.O. Box, CH-4002 Basel, Switzerland and [4]Institut für klinische Pharmakologie, Uniklinik Frankfurt, Theodor-Stern-Kai 7, D-60590 Frankfurt a. M., Germany

Email: Helge B. Bode* - h.bode@bio.uni-frankfurt.de

*Corresponding author

[§]Bioactivity testing

NMR-data of szentiamide (1)

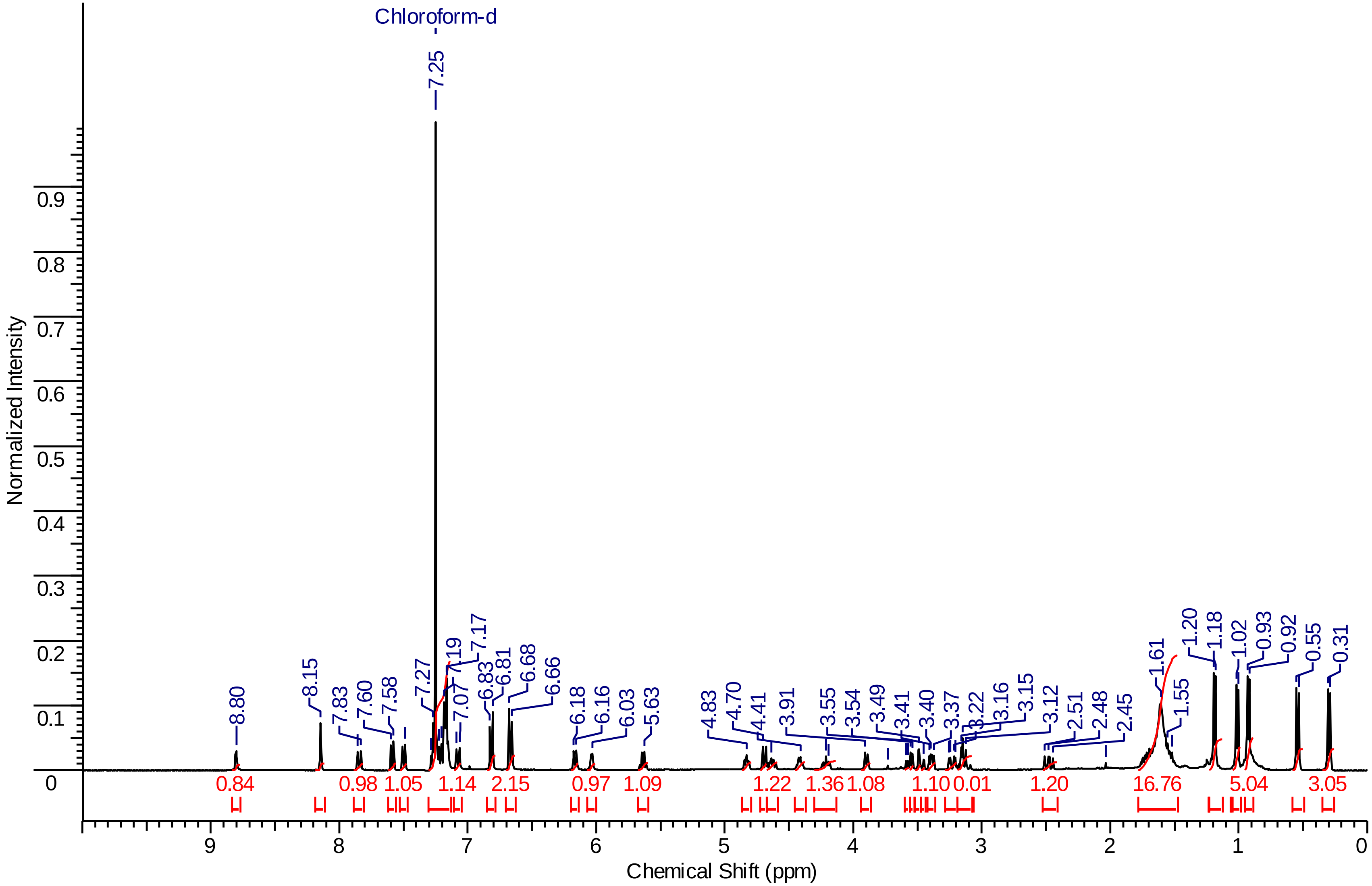

Figure S1: NMR-spectra of the purified “natural” Szentiamide (400 MHz; $CDCl_3$).

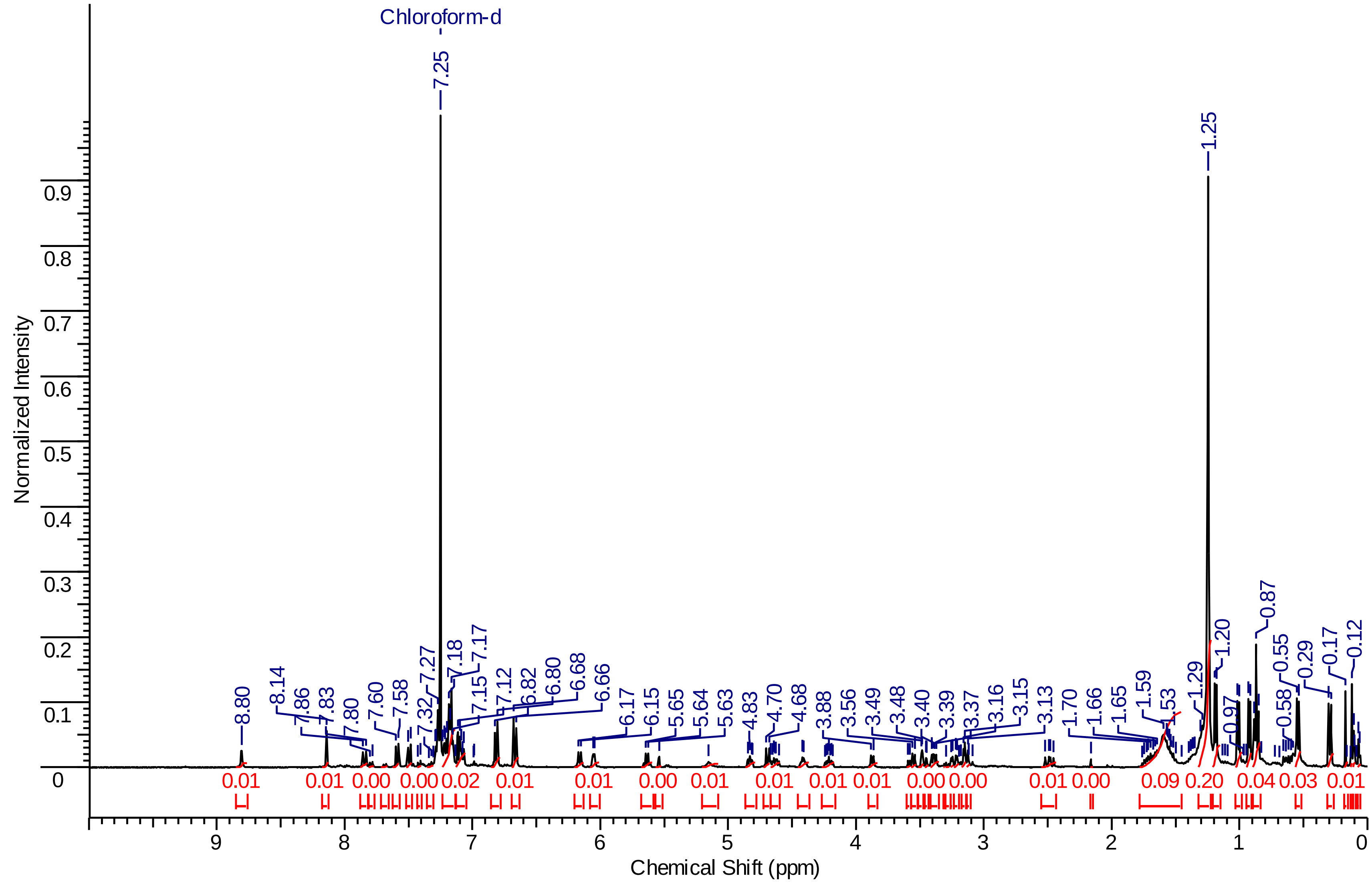

Figure S2: NMR-spectra of the purified "synthetic" Szentiamide (400 MHz; $CDCl_3$).

Table S1: NMR data of the "natural" and "synthetic" Szentiamide

	"natural" Szentiamide		"synthetic" Szentiamide	
	1H	Comment	1H	Comment
$CDCl_3$/Indol-CH/ Val-NH	7.12-7.3	16 H, m	7.12-7.3	20 H, m
Formyl	8.15	1H, s, sharp	8.14	1H, s, sharp
Leu-CH alpha	4.42	1H, m	4.41	1H, m
Leu-CH_2 beta/ Leu-CH gamma	1.48-1.77	17H, m	1.45-1.76	12H, m
Leu-CH_3	1.01	3H, d (*J*=6.0 Hz)	1.02	3H, d (*J*=6.0 Hz)
Leu-CH_3	0.93	5H, d (*J*=6.0 Hz)	0.92	3H, d (*J*=6.0 Hz)
Leu-NH	6.04	1H, d (*J*=4.5 Hz)	6.05	1H, d (*J*=4.5 Hz)
Phe-CH alpha	4.83	1H, m	4.83	1H, m
Phe-CH beta	3.52-3.61	1H, m (1:2)	3.54-3.59	1H, m (1:2)
Phe-CH beta	3.43-3.51	1H, m (2:1)	3.45-3.49	1H, m (2:1)
Phe-NH	6.17	1H, d (*J*=8.0 Hz)	6.16	1H, d (*J*=7.5 Hz)
Thr-CH alpha	4.69	1H, d (*J*=10.0 Hz)	4.69	1H, d (*J*=10.0 Hz)
Thr-CH beta	5.64	1H, q (*J*=6.5 Hz)	5.64	1H, q (*J*=6.5 Hz)
Thr-CH_3/ Val-CH beta	1.19	4H, d (*J*=6.5 Hz)	1.19	4H, d (*J*=6.5 Hz)
Thr-NH	7.85	1H, d (*J*=10.0 Hz)	7.85	1H, d (*J*=10.0 Hz)
Trp-CH alpha	4.6-4.67	1H, m	4.61-4.67	1H, m
Trp-CH_2 beta	3.07-3.19	2H, m	3.13-3.26	3H, m
Trp-NH	7.08	1H, d (*J*=8.5 Hz)	7.1	3H, m
Indol-CH	7.59	1H, d (*J*=8.0 Hz)	7.59	1H, d (*J*=8.0 Hz)
Indol-CH	7.5	1H, d (*J*=8.0 Hz)	7.49	1H, d (*J*=8.0 Hz)
Indol-NH	8.8	1H, s, broad	8.8	1H, s, broad
Tyr-CH alpha	4.21	1H, m	4.21	1H, m
Tyr-CH aromat	6.82	2H, d (*J*=8.5 Hz)	6.81	2H, d (*J*=8.5 Hz)
Tyr-CH aromat	6.67	2H, d (*J*=8.5 Hz)	6.67	2H, d (*J*=8.5 Hz)
Tyr-CH beta	3.23	1H, dd (*J*=15.0 Hz; *J*=4.0 Hz)	3.23	1H, dd (*J*=15.0 Hz; *J*=4.0 Hz)
Tyr-CH beta	3.39	1H, dd (*J*=9.0 Hz; *J*=5.0 Hz)	3.39	1H, dd (*J*=9.0 Hz; *J*=5.0 Hz)
Tyr-NH	3.9	1H, d (*J*=8.0 Hz)	3.87	1H, d (*J*=8.0 Hz)
Val-CH alpha	2.48	1H, dd (*J*=13.5 Hz; *J*=11.5 Hz)	2.49	1H, dd (*J*=15.0 Hz; *J*=11.5 Hz)
Val-CH_3	0.54	3H, d (*J*=7.0 Hz)	0.55	3H, d (*J*=7.0 Hz)
Val-CH_3	0.3	3H, d (*J*=7.0 Hz)	0.3	3H, d (*J*=6.5 Hz)
